# B Epitope Multiplicity and B/T Epitope Orientation Influence Immunogenicity of Foot-and-Mouth Disease Peptide Vaccines

**DOI:** 10.1155/2013/475960

**Published:** 2013-12-03

**Authors:** Esther Blanco, Carolina Cubillos, Noelia Moreno, Juan Bárcena, Beatriz G. de la Torre, David Andreu, Francisco Sobrino

**Affiliations:** ^1^Centro de Investigación en Sanidad Animal (CISA-INIA), Valdeolmos, 28130 Madrid, Spain; ^2^Departament de Ciències Experimentals i de la Salut, Universitat Pompeu Fabra, 08003 Barcelona, Spain; ^3^Centro de Biología Molecular, “Severo Ochoa” (CSIC-UAM), Cantoblanco, 28049 Madrid, Spain

## Abstract

Synthetic peptides incorporating protective B- and T-cell epitopes are candidates for new safer foot-and-mouth disease (FMD) vaccines. We have reported that dendrimeric peptides including four copies of a B-cell epitope (VP1 136 to 154) linked to a T-cell epitope (3A 21 to 35) of FMD virus (FMDV) elicit potent B- and T-cell specific responses and confer protection to viral challenge, while juxtaposition of these epitopes in a linear peptide induces less efficient responses. To assess the relevance of B-cell epitope multivalency, dendrimers bearing two (B_2_T) or four (B_4_T) copies of the B-cell epitope from type O FMDV (a widespread circulating serotype) were tested in CD1 mice and showed that multivalency is advantageous over simple B-T-epitope juxtaposition, resulting in efficient induction of neutralizing antibodies and optimal release of IFN**γ**. Interestingly, the bivalent B_2_T construction elicited similar or even better B- and T-cell specific responses than tetravalent B_4_T. In addition, the presence of the T-cell epitope and its orientation were shown to be critical for the immunogenicity of the linear juxtaposed monovalent peptides analyzed in parallel. Taken together, our results provide useful insights for a more accurate design of FMD subunit vaccines.

## 1. Introduction

Foot-and-mouth disease (FMD) is a highly infectious disease of cloven-hoofed animals, admittedly the most important livestock disease in terms of economic impact [1, 2]. In many areas of the world (Africa, Asia, and to some extent South America) where it remains endemic, FMD severely handicaps access to international meat markets [3]. FMD control in endemic regions is implemented mainly by using chemically inactivated whole-virus vaccines whose production (large-scale growth of virulent FMD virus (FMDV), virus inactivation, antigen concentration, and purification) has remained largely the same for decades [4, 5]. This practice poses serious biosafety concerns, as the risk of virus release during vaccine production is well documented [4, 6]. Additional shortcomings of current FMD vaccines include the need for a cold chain to preserve virus stability and the difficulty of serological distinction between infected and vaccinated animals [4]. These concerns, along with the severe trade restrictions applied in any vaccination campaign, have led FMDV-free countries to adopt nonvaccination policies in the event of an FMD outbreak. Such policies have involved massive slaughtering of infected or suspected (contact) herds, as well as strict limitations on farm animal movements, all of which have raised significant controversies [7, 8].

In this context, peptide-based approaches appear to be an attractive strategy for FMD vaccine development. Peptide vaccines offer several advantages over other types of vaccines such as (i) lack of infectious agent, which ensures absolute safety, (ii) accurate molecular delineation of the immunogen, which allows to exclude detrimental sequences present in full-length antigens or other pathogen-related molecules, and permits easy differentiation of infected from vaccinated animals (referred as DIVA vaccines), (iii) easy access and scale up by peptide synthesis methods, and (iv) uncomplicated transport and storage [9, 10]. However, despite their promises candidates for next-generation vaccines and peptides are typically poorly immunogenic.

In the case of FMD, the main antigenic sites recognized by B lymphocytes have been identified at defined structural motifs exposed on the surface of the FMDV capsid, whose amino acid sequences accumulate variations among different serotypes [11, 12]. A continuous, immunodominant B-cell site located in the GH loop, around positions 140 to 160 of capsid protein VP1, has been widely used as an immunogenic peptide [13]. However, the protection against FMD conferred to natural hosts by linear peptides spanning the GH loop of VP1 is limited [13, 14]. Although it is generally accepted that protective immunity to FMDV is mostly due to neutralizing antibodies, a T-cell response is also necessary for effective immunity [15]. We have reported that inclusion of a specific T-cell epitope located in residues 21 to 35 of FMDV NS protein 3A [16], juxtaposed in a linear fashion to the above-mentioned B-cell epitope, affords significant reduction in virus excretion and clinical scores after challenge [17]. This monomeric peptide B-T, however, did not afford full protection in challenged pigs.

In order to enhance the effectiveness of presentation of the above B- and T-cell epitopes to the immune system, a multimerization approach consisting of a dendrimeric peptide bearing four copies of the B-cell epitope and one of the Th epitope was explored. The branched construct, generically known as B_4_T, elicited high titers of FMDV-neutralizing and IgA antibodies in both pig and outbred mice (CD1 strain) activated T-cells and induced IFN*γ* release. The dendrimer performed as an effective peptide vaccine conferring solid protection in swine against type C FMDV challenge [18].

With a view to extending these proof-of-concept results to epidemiologically relevant FMDV serotypes, we have designed and produced new B_4_T constructs harboring sequences from type O FMDV (currently the most widespread serotype) [3, 19] as B-cell epitopes. To assess the relevance of B-cell epitope multivalency, constructs bearing two (B_2_T) or four (B_4_T) copies of the B-cell epitope were tested in CD1 mice, showing that multivalency is advantageous over simple B-T-epitope juxtaposition but also that bivalent B_2_T constructions elicit similar or even better humoral and cellular specific responses than tetravalent ones [20]. Herein we show that epitope density is essential for inducing an efficient humoral immune response, including neutralizing antibodies, but also for optimal release of IFN*γ*, a relevant antiviral cytokine. In addition, we evaluate the orientation of T-relative to B-cell epitopes in juxtaposed monovalent constructs in terms of efficient T-B cell cooperation and induction of cellular response. Taken together, our results provide useful insights for a more accurate design of subunit vaccines.

## 2. Materials and Methods

### 2.1. Peptides

Peptides reproducing the B [VP1(136–150)] and T [3A(21–35)] epitopes of type O FMDV (O-UKG 11/01) in different arrangements are shown in Table 1. Linear B, T, BT and TB peptides were made by Fmoc solid phase synthesis as previously described [17]. Bi- and tetravalent constructs B_2_T and B_4_T were made by thioether ligation protocols as detailed in Monso et al. [20] and Cubillos et al. [18], respectively. All six peptides were purified by reverse phase HPLC to >95% purity and satisfactorily characterized by MALDI-TOF mass spectrometry.

### 2.2. Virus

A virus stock derived from serotype O-UKG 11/01 (supplied by The Pirbright Institute, UK) by two amplifications in IBRS-2 cells, which maintained the consensus sequences at the capsid protein region, was used in this study.

### 2.3. Mice and Immunization

The immune response induced by the various peptide constructions on Table 1 was assessed in outbred Swiss mice (Swiss ICR-CD1, Harlan Laboratories). 5- to 6-week-old female mice in groups of 6 to 9 were maintained under standard housing conditions in the CBMSO Animal Care unit. Mice were immunized subcutaneously at days 0 and 18 with 100 *μ*g of each peptide emulsified in Montanide ISA 50V2 (Seppic, France) and sacrificed at day 39. Two groups of 4 mice each were immunized as controls with either adjuvant or PBS. Blood samples were collected by tail bleeding before priming (day 0) and at days 14, 20, and 39 (sacrifice). All experimental procedures were conducted in accordance with protocols approved by the CBMSO ethical committee.

### 2.4. Virus Neutralization Test (VNT)

Virus neutralization activity was determined in sera by a standard microneutralization test performed in 96-well plates by incubating serial twofold dilutions of each serum sample with 100 50% tissue culture infective dose (TCID50) of FMDV O-UK/01, for 1 h at 37°C. The remaining viral activity was determined in 96-well plates containing fresh monolayers of BHK-21 cells. Endpoint titers were calculated as the reciprocal of the final serum dilution that neutralized 100 TCID50 of FMDV (O-UK/01) in 50% of the wells [22].

### 2.5. Detection of Specific Anti-FMDV Antibodies By ELISA

Total antibodies against FMDV were examined by ELISA in serum samples collected at day 39. Briefly, Maxisorp 96-well plates (Nunc) were coated with sucrose gradient-purified 140's FMDV O UKG 11/2001 particles in PBS overnight at 4°C (0.1 *μ*g/well). Then, wells were washed with PBS and blocked with 5% skimmed milk in PBS for 2 h at 37°C. Duplicate threefold dilution series of each serum sample were prepared, starting at 1/100 and made up at 50 *μ*L. Preimmune sera from peptide-immunized mice and sera from nonimmunized animals were used as negative controls. Specific antibodies were detected with horse radish peroxidase-(HRP-) conjugated goat anti-mouse IgG (Zymed, Invitrogen), diluted 1/3000. Colour development was obtained after addition of 100 *μ*L/well of the soluble substrate 3,3′,5,5′-tetramethylbenzidine (TMB, Sigma) and stopped by an equal volume of 1 M H_2_SO_4_. Plates were read in a Fluostar Omega microplate reader at 450 nm. In this assay, it was found that the point on the titration curve corresponding to an A_450_ of 1.0 invariably fell on the linear part of the curve. Antibody titers were therefore expressed as the reciprocal of the last dilution calculated by interpolation to give an absorbance of 1 above background.

### 2.6. Detection of Isotype-Specific Anti-FMDV Antibodies by ELISA

FMDV-specific IgG1, IgG2a, IgG2b (in sera) and IgA (in sera and nasal swabs) were measured using a modification of the ELISA described above. 100 *μ*L volumes were used throughout. In the case of nasal swabs, two consecutive incubations with sample were performed before adding the commercial antibody (Sigma) to mouse IgA, in order to increase the sensitivity of the assay. Isotyping ELISA was performed using a goat anti-mouse specific for mouse isotypes and as secondary a rabbit anti-goat HRP-IgG (both by Sigma). Plates were developed by TMB before the stop solution (0.16 M H_2_SO_4_) was added and read at 450 nm as above. Endpoint titers were expressed as the reciprocal log⁡_10_ of serum dilutions giving the absorbance recorded in the control wells (serum collected day 0) plus twice standard deviation. These data were calculated by interpolation. IgA titers in nasal swabs were expressed as the absorbance at a dilution of 1 : 10.

### 2.7. IFN-*γ* Detection by ELISPOT

A mouse IFN-*γ* ELISPOT reagent kit was used according to manufacturer's instructions (BD Biosciences). Briefly, after red blood cell lysis, (1 and 8 × 10^5^) splenocytes were distributed in triplicate wells of Immobilon-P hydrophobic PVDF 96-well plates (Millipore) previously coated with an anti-mouse IFN-*γ* antibody, and then blocked with RPMI 1640 (Gibco) supplemented with glutamine, penicillin/streptomycin, and 10% inactivated FSB. Cells were stimulated with either autologous peptide at a concentration of 0.4 *μ*g/well or with 1 *μ*g/well of sucrose gradient-purified FMDV (O UKG 11/2001). Triplicate wells with 8 × 10^5^ cells without peptide were used to estimate nonspecific activation. As positive control, triplicate wells with 8 × 10^5^ cells were stimulated with phytohemagglutinin (Sigma) at 10 *μ*g/mL per well. After 48 h at 37°C, 5% CO_2_ and 95% relative humidity, plates were washed and incubated with a biotinylated anti-mouse IFN-*γ* antibody followed by HRP-streptavidin. Antibody binding was visualized by the substrate 3-amino-9-ethylcarbazole. The frequency of peptide- and virus-specific T cells present in the responding population was expressed as the mean number of spot-forming cells (SFC) counted in stimulated wells per 10^6^ splenocytes.

### 2.8. Statistical Analyses

Statistical significance between three and more groups was calculated with one-way ANOVA, followed up by multiple comparison post-tests (Bonferroni or Tukey test depending on the sample sizes compared). Statistical tests were performed using Prims software (GraphPad Software). *P* values are depicted as follows: *P < 0.05; **P < 0.01; and ***P < 0.001.

## 3. Results

### 3.1. B and T Epitope Relative Positioning and B Epitope Multiplicity Are Both Essential for Anamnestic Humoral Responses against FMDV

Various presentations of the FMDV B and T epitopes (Table 1) were used to address issues such as (i) the effect of a T epitope on immunogenicity or the ideal relative orientation of juxtaposed B and T epitopes displayed in linear peptides and (ii) how B epitope multiplicity bore on the immune response of branched constructs B_2_T and B_4_T. In contrast to B cell epitopes, for which multiple presentation has a favourable effect on the immune response (see below), T-cell stimulation and the ensuing enhancement in immune response are achievable by a single copy of the epitope. Upon this rationale, T_2_B and T_4_B architectures, though synthetically feasible, were predicted as unpromising hence disregarded for this study.

**Figure 1:**
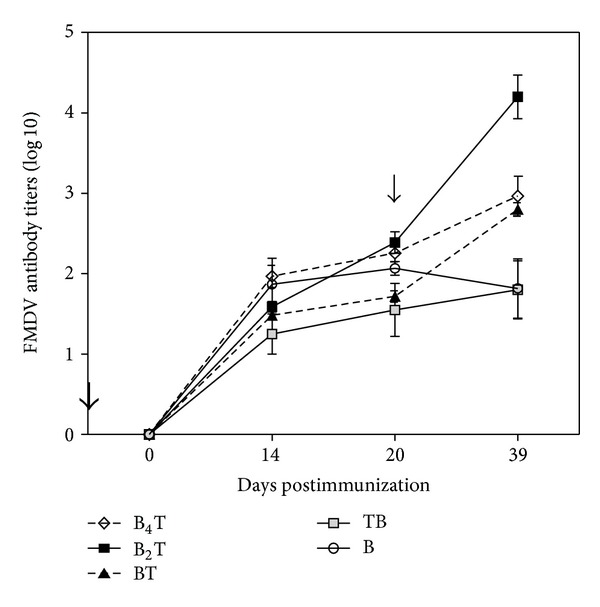
Time course study of antibody responses to FMDV in sera analyzed by ELISA. Specific antibody titers against FMDV (O UKG 11/2001) measured by ELISA in serum samples collected at days 0, 14, 20, and 39 postimmunization. Antibody titers were expressed as the reciprocal log⁡_10_ of the last dilution calculated by interpolation to give an absorbance of 1 above background. Each point corresponds to the geometric mean of a least two determinations. In all cases the standard deviation was <1.0. Arrows indicate immunization (first dose and boost) dates.

Specific anti-FMDV antibodies were determined by ELISA in the sera of peptide-immunized mice collected at days 0, 14, 20, and 39. As expected, no anti-FMDV antibodies were detected in animals inoculated with peptide T (data not shown). On the contrary, all peptides in Table 1 containing the B cell epitope elicited significant (>1log⁡_10_) anti-FMDV titers after a single dose (Figure 1). However, clear differences among the peptides were recorded in the humoral response induced by the booster injection. Thus, only the branched B_4_T and B_2_T constructs and monomeric BT, with the B at the N-terminus and followed by the T-cell epitope sequence, were able to induce secondary antibody responses after re-exposure to immunogen. This suggests that placing the B-cell epitope at the N-terminal to the T-cell epitope is essential for effective cooperation, particularly for specific B-cell memory induction.

Regarding the magnitude of humoral response, the titre of specific antibodies against FMDV was highest in mice immunized with dendrimeric B_2_T (average titer 4.2 ± 0.6 SD), well above (about 1log⁡) those of tetravalent B_4_T and linear BT, both of which induced similar responses.

### 3.2. Multiple Presentation of B-Cell Epitopes in Dendrimeric Constructions Significantly Enhances the Generation of Specific Virus Neutralizing Antibodies

**Figure 2:**
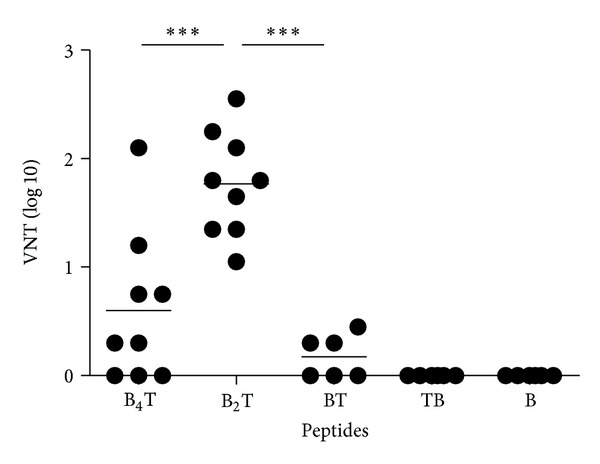
Antibody responses to FMDV in sera analyzed by neutralization assay at day 39 postimmunization. The VNT titers determined in each group of mice were expressed as the reciprocal (log⁡_10_) of the last dilution able to neutralize 100 TICD_50_ of homologous virus. Animals with VNT ≥ 1 are considered seropositive. Each symbol represents the value for an individual animal. Horizontal lines indicate the mean value for each group of animals. Statistical differences between B_2_T group and the others are indicated by *P* values.

Clear and significant differences in the virus neutralizing antibody titers (VNT) were found after immunization with either dendrimeric or linear peptides. The highest VNT was detected in mice immunised with bivalent B_2_T (Figure 2). All the animals within this group (*n* = 9) had significant VNT (>1log⁡). The magnitude of neutralizing antibody response in B_2_T group was even significantly higher than that induced by the other dendrimeric peptide assayed, the B_4_T construction (*P* < 0.001). These statistical differences in the neutralizing antibody titers induced by the peptide B_2_T were also found relative to the monomeric constructions (BT, TB, and B) (Figure 2).

Regarding VNT induced by the monomeric peptides (BT, TB, and B), they were only detectable in the first (BT-immunized) group, albeit below the significance threshold, (3 out of 6 immunized mice). These results suggest that multimeric presentation of B-cell epitopes in the dendrimers, as well as the presence of a T-cell epitope in an adequate orientation in linear peptides, enhance the induction of VN antibodies. Again, no anti-FMDV neutralizing antibodies were detected in animals inoculated with peptide T (data not shown).

### 3.3. Incorporation of the T-Cell Epitope Is Required to Elicit IgG2 Responses

**Figure 3:**
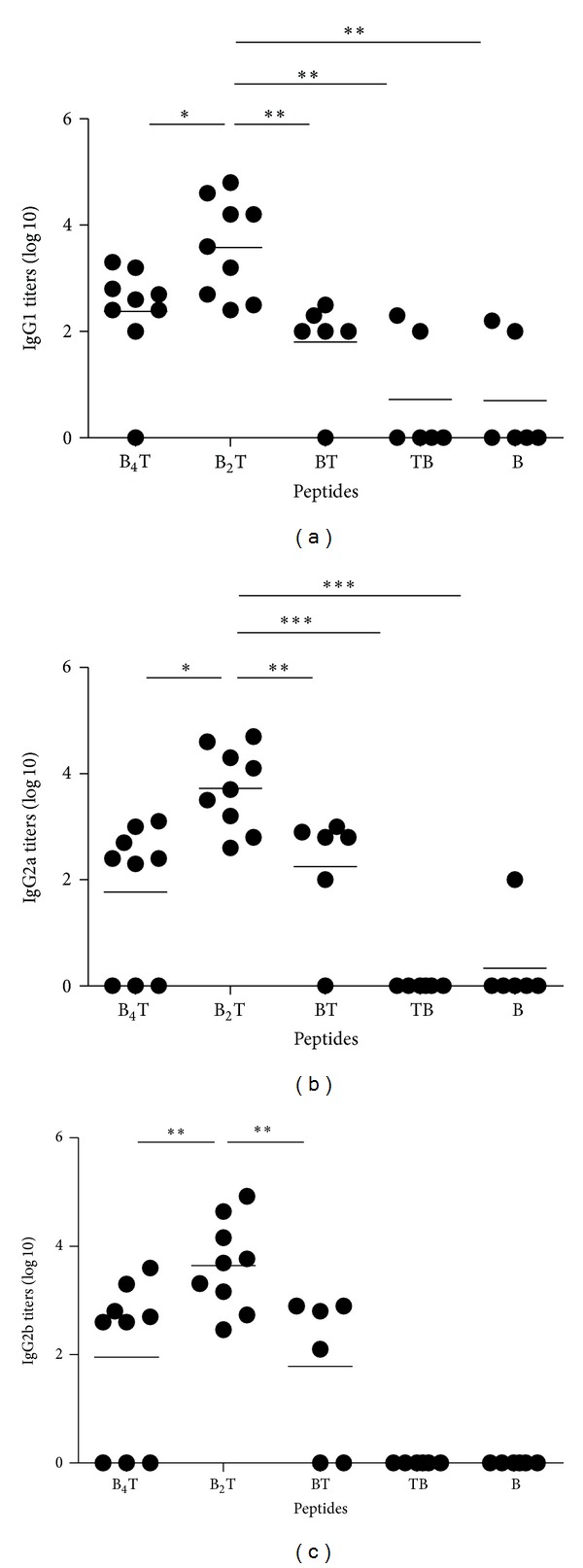
Isotype-specific antibody (IgG1 and IgG2a/G2b) responses to FMDV in sera analyzed by ELISA at day 39 postimmunization. Endpoint titers were expressed as the reciprocal log⁡_10_ of serum dilutions giving the absorbance recorded in the control wells (serum collected day 0) plus twice standard deviation. Each symbol represents the value of individual mouse. Horizontal lines indicate the mean value for each group of animals.

We next investigated the ability of the different peptides delivered subcutaneously to induce specific isotype IgG1 and IgG2a/IgG2b antibodies. As shown in Figure 3, B_2_T was the construction that induced significantly higher levels of the anti-FMDV antibodies of the three isotypes. In addition, isotyping of the anti-FMDV antibodies revealed a Th1/Th2 balanced profile when the peptides harbour the T-cell epitope in a suitable orientation. Thus, animals immunized with peptides TB and B did not show specific IgG2b titers and only one of the mice immunized with peptide B elicited IgG2a titers. These results suggest that class-switching (production of IgG2a and IgG2b) of the FMDV antibodies induced by linear peptides requires the cooperation of CD4+ lymphocytes. On the other hand, multimerization of the B-cell epitope in the T-cell epitope-containing divalent dendrimers results in improved titers of the three IgG isotypes.

### 3.4. Multiple Presentation of the B-Cell Epitope Enhances Systemic and Local Specific IgA Responses

**Figure 4:**
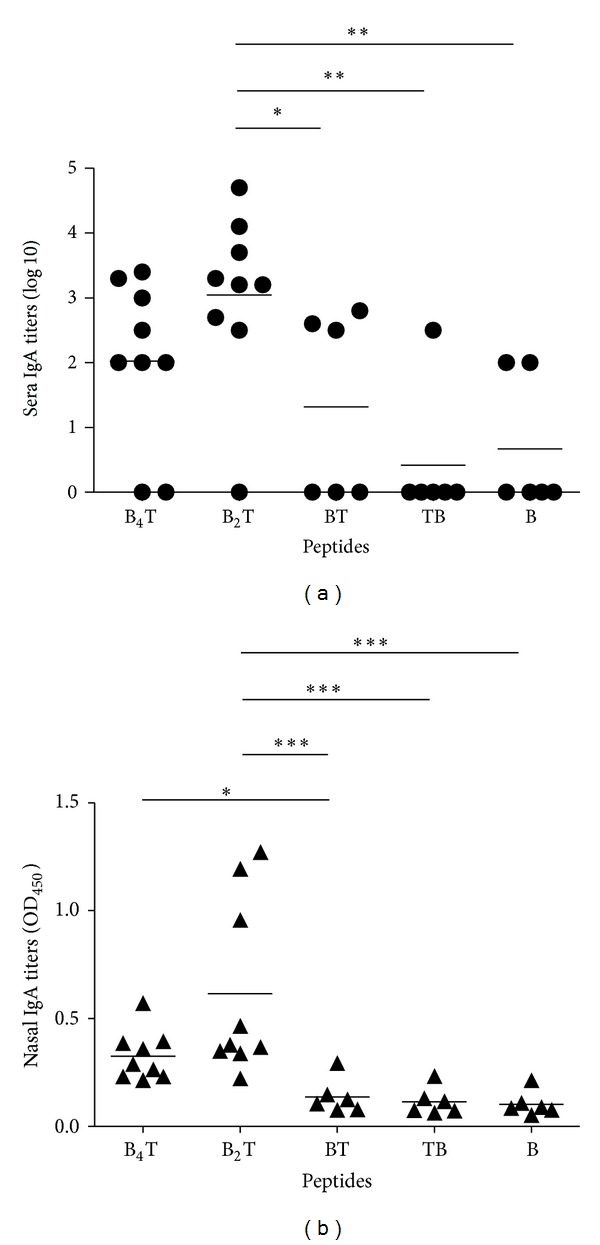
Systemic (serum) and local (nasal) IgA antibody responses to FMDV determined by ELISA at day 39 postimmunization. (a) IgA antibody titers against FMDV. Endpoint titers were expressed as described in Figure 3. Each symbol represents the value for an individual mouse. Horizontal lines indicate the mean value for each group of animals. (b) IgA antibody titers against FMDV determined in nasal fluids. Titers are expressed as the OD at 450 nm value obtained using a 1 : 10 dilution.

To investigate the capacity of the peptides assessed to elicit systemic and local specific IgA responses, serum samples and nasal fluids were analysed by an IgA-specific ELISA (Figure 4). The higher IgA responses, in most of the immunized mice, were elicited by multimeric peptides (B_4_T and B_2_T). Statistical differences were found between FMDV-specific IgA antibodies titers elicited by peptides B_2_T and B_4_T and the three monomeric peptides, but not between both dendrimeric constructions (Figure 4). Thus, multimerization of the B-cell epitope results in an improvement of both sera and nasal specific IgA titers.

### 3.5. Multiple Presentation of the B-Cell Epitope Improves T-Cell Mediated IFN-*γ* Release

**Figure 5:**
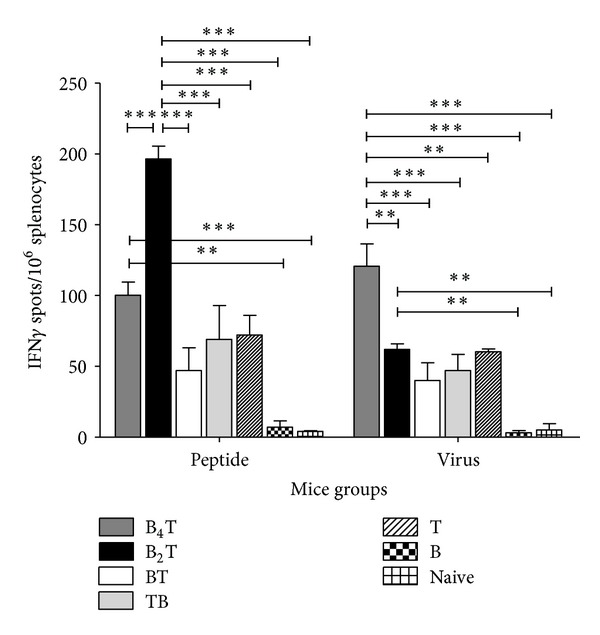
Frequency of peptide- and virus-specific IFN*γ*-producing cells in the spleen of immunized mice. IFN*γ*-producing cells were measured by ELISPOT assay in four animals from each group. Cells were stimulated with peptide (white bars) or FMDV (grey bars). Data are presented as the mean for four mice; error bars represent standard deviation.

Freshly collected splenocytes were stimulated *in vitro* with either the immunizing peptide or FMDV. After 48 h incubation, cells were harvested and their activation was analyzed by an IFN-*γ* ELISPOT assay. All the synthetic peptides including that corresponding to the T-cell epitope were able to induce significant release of this cytokine after *in vitro* recall with the corresponding antigens, peptide and virus (Figure 5). The highest release of IFN-*γ* in response to *in vitro* recall with FMDV was induced by B_4_T, followed by B_2_T construction. However, when the corresponding peptides were used as *in vitro* stimuli, IFN-*γ* release was higher induced by B_2_T peptide. Differences with monomeric peptides were statistically significant between B_2_T and/or B_4_T versus BT, TB, and B, after stimulation with peptide and virus, respectively. Only 3 out of 6 animals immunized with linear peptides were responders in the ELISPOT assay, while all the dendrimeric peptide-immunized mice released detectable quantities of IFN-*γ*.

## 4. Discussion

The development of successful peptide vaccines has been limited mainly by difficulties associated with *in vivo* stability, poor immunogenicity of linear peptides, and by the MHC polymorphism of the host species [23, 24]. However, recent advances on the requirements for induction and maintenance of immune responses, as well as on the pharmacokinetics of peptides, have provided new strategies to enhance both peptide immunogenicity and stability, and thus returned peptide-based technologies to the forefront of vaccine design [9, 10].

The epitope density of a single antigen molecule is convincingly regarded as a self/non-self-discriminator of humoral responses [25, 26]. Usually, repetitive viral surface antigens of high epitope density induce efficient direct cross-linking of the surface Ig receptors of immature B cells, whereas monomeric antigens of low epitope density rather induce apparent B-cell tolerance [25, 26]. This effect, together with the facilitation of antigen internalization in antigen presenting cells (APC) such as dendritic cells, has been proposed as an explanation for the ability of multimeric presentations to trigger an efficient antibody response [27]. In line with this multimerization approach, we previously reported full protection against FMDV following vaccination of pigs with dendrimeric peptides (B_4_T) displaying B- and T-cell epitopes of FMDV serotype C [18]. However, the same B and T epitopes juxtaposed colinearly elicited only partial protection, highlighting the relevance of T-cell epitope insertion and multimeric presentation [17].

To extend this proof of concept to epidemiologically relevant FMDV serotypes, in the present study we have designed new linear and dendrimeric peptides bearing type O FMDV B-cell epitope sequences (from the GH loop of VP1), and the same T-cell epitope used in previous studies [16–18], which is highly conserved among FMDV serotypes [28]. We have explored the contribution to immunogenicity of T-cell epitope incorporation in type O FMDV linear monomeric peptides, by analyzing how the lack of such T-cell epitope (peptide B) or the N- or C-terminal attachment of the T to B epitope (peptides BT an TB) affects the immune response. Our results show that T-cell epitope incorporation in monomeric peptides is essential for an efficient induction of antibodies, including development of anamnestic response and anti-FMDV neutralizing antibodies. Furthermore, data for the linear peptides support that incorporation of the T-cell epitope in the proper orientation relative to the B epitope (peptide BT) is essential for the induction of effective T-cell help. Thus, either the lack of T-cell epitope or its positioning at the N-terminus of the B-cell epitope abrogates both the secondary humoral response after re-exposure to immunogen (Figure 1) and the induction of specific neutralizing antibodies (Figure 2). Class-switching (production of IgG1, IgG2a, and IgG2b) is a process that requires the contribution of CD4+ lymphocytes [4]. Immunization with TB or B peptides did not elicit specific IgG2 antibodies (Figure 3). Therefore, adequate orientation of the T-cell epitope in the monomeric peptides results in both enhanced humoral response and in a bias towards Th-1 type immunity. In this regard, it is worth recalling that in both linear constructions the B and T epitopes are juxtaposed, not separated by any connecting unit. These two different epitope arrangements define in fact rather different structural patterns, in spite of identical amino acid composition. One should therefore not exclude that such different molecular positioning underlies the different processing of BT and TB peptides, altering the T-cell epitope binding to MHC or the recognition of the B-cell epitopes by B cells.

From these results we can conclude that, in CD1 mice, the immune response to this particular B-cell epitope in the GH loop of VP1 is T-cell-dependent, requiring an efficient cooperation of specific CD4+ lymphocytes. This result is consistent with studies in cattle by Juleff et al. [29], who found that CD4 depletion substantially inhibited antibody responses to GH loop peptide VP1(135–156), indicating that responses to this particular site in cattle were T-cell-dependent. Interestingly, while N-terminal positioning of the T-cell epitope (TB peptide) failed to elicit anti-FMDV antibodies, cellular responses were evoked in immunized mice (Figure 5). This suggests that T-cell epitope orientation is only relevant for efficient B-T cooperation, but does not significantly affect *in vivo* priming of peptide-specific T-cells upon *in vitro* recall to release IFN*γ* in response to such stimulation (Figure 5). In this regard, some questions still remain to be addressed. For example, it would be interesting to determine more precisely how effector and memory T cells are activated after immunization with the dendrimeric peptide, and which cell subset (T, NKT, NK, B, or professional APCs) is responsible for IFN*γ* production. These analyses would likely provide some explanation for the differences between tetra- and bivalent constructions in their IFN*γ* response to virus or peptide. This study has also confirmed our previous findings with type C FMDV dendrimeric constructions. Results with B_4_T and B_2_T dendrimers support that multiple presentation of the B-cell epitope is advantageous over simple juxtaposition for induction of humoral and cellular immune responses [17, 18]. Furthermore, we have shown that B_2_T, a downsized version of B_4_T with only two copies of the B-cell epitope, elicits similar or even better responses [20]. We still do not have an explanation for this somewhat unexpected observation, although one could conjecture that differences in epitope multiplicity/display might reduce B-cell receptor cross-linking efficiency and eventually lower B_4_T immunogenicity relative to B_2_T.

Dendrimeric and linear peptides also performed differently with regard to IgA antibody titers. As most pathogens, FMDV included, enter the body through mucosal surfaces [30], mucosal IgA responses are an effective, front-line way of fighting infection. There is early evidence of the role of specific IgA in protection against FMDV [31], pointing out that effective stimulation of mucosal IgA response would be crucial for the successful development of FMDV vaccines [17, 32, 33]. Consistently with our previous results [18, 20], FMDV type O bi- and tetravalent constructions induced high mucosal responses by parenteral administration (Figure 4(b)), while none of the monomeric peptides were able to induce nasal FMDV IgA titers. The natural pathway of mucosal immune induction involves direct delivery of the immunogen to a mucosal surface and local processing of the antigen in specialized aggregates of lymphoid tissue, termed mucosal inductive sites [34, 35]. Stimulated lymphocytes then migrate to the corresponding mucosal surface where antigen-specific IgA and IgG are locally produced, and specific T cells reside to protect that mucosal surface from pathogen attack [35]. In contrast to this natural induction pathway, B_4_T and B_2_T dendrimers can induce high mucosal immune responses by parenteral administration. Alternative, nonmutually exclusive mechanisms can be invoked for the production of secretory antibodies after parenteral administration of the antigen, including (i) direct diffusion of soluble or phagocytosed antigens to mucosa-associated lymphoid tissue and/or (ii) activation of antigen-presenting cells at draining lymph nodes, which then migrate to mucosa-associated lymphoid tissue [35, 36]. The dendrimeric peptides are more stimulatory for the mucosal immune system than monomeric peptides probably due to better targeting to dendritic cells. Taken together, these results confirm dendrimeric peptides as good candidates for future mucosal vaccines, able to overcome some of the main problems associated with mucosal administration (local degradation, physical ejection of the vaccine, etc.) [34].

In addition to confirming that multimerization is an important strategy in the triggering of a humoral response, herein we have shown it can also improve IFN-*γ* release by primed splenocytes after *in vitro* recall. Thus, higher IFN-*γ* release levels were observed after immunization with dendrimeric (particularly B_4_T) than with linear peptides. IFN*γ*, a key cytokine produced primarily by NK and T cells, facilitates host defense against intracellular pathogens [23]. CD4+ T cell activation by DCs triggers their differentiation along two distinct and mutually exclusive pathways: (i) Th1-type CD4+ T cells essentially produce IFN-*γ* and TNF-*α*, which assist in the elimination of intracellular pathogens both directly (cytokine responses) and indirectly via their support to macrophage activation and CD8+ T cell differentiation and (ii) Th2-type CD4+ T cells essentially produce IL-4, IL-5, and IL-13 which are directly involved in the defense against extracellular pathogens such as helminths [26]. Enhanced uptake by DCs of high epitope density antigens, such as dendrimeric peptides and/or their mechanism of internalization [27, 37], might explain upregulated IFN-*γ* production. In contrast, processing of linear peptides by other APCs (macrophages, neutrophils, and B cells), not so efficient in activation of Th1 responses, could explain the lower IFN-*γ* release levels in mice immunized with these peptides.

Finally, the fact that a bivalent peptide is more immunogenic than its tetravalent congener has also important implications for developing cost-effective FMDV peptide vaccines. Production of B_4_T required exhaustive purification and even so yielded a not-quite-homogeneous end product [18], whereas the simpler design and improved conjugation chemistry of the B_2_T constructs translated into significantly improved yields, purity, and hence much lower production costs [20]. At the time of this writing, it seems clear that the B_2_T construct shows undeniable advantages as a candidate for a peptide-based FMDV vaccine.

## 5. Conclusions

The above results highlight the relevance of adequately selecting specific B- and T-cell epitopes to be included in a peptide vaccine, as well as the importance of B/T cell epitope relative positioning for successful eliciting of an immune response. In our FMDV model, B-cell epitope multivalency by means of dendrimeric constructions is shown to improve immunogenicity, including induction of mucosal IgA, relative to mere linear juxtaposition of B- and T-cell epitopes. Moreover, bivalent dendrimeric constructions elicit similar or even better immune responses than tetravalent ones.

## Figures and Tables

**Table 1:** 

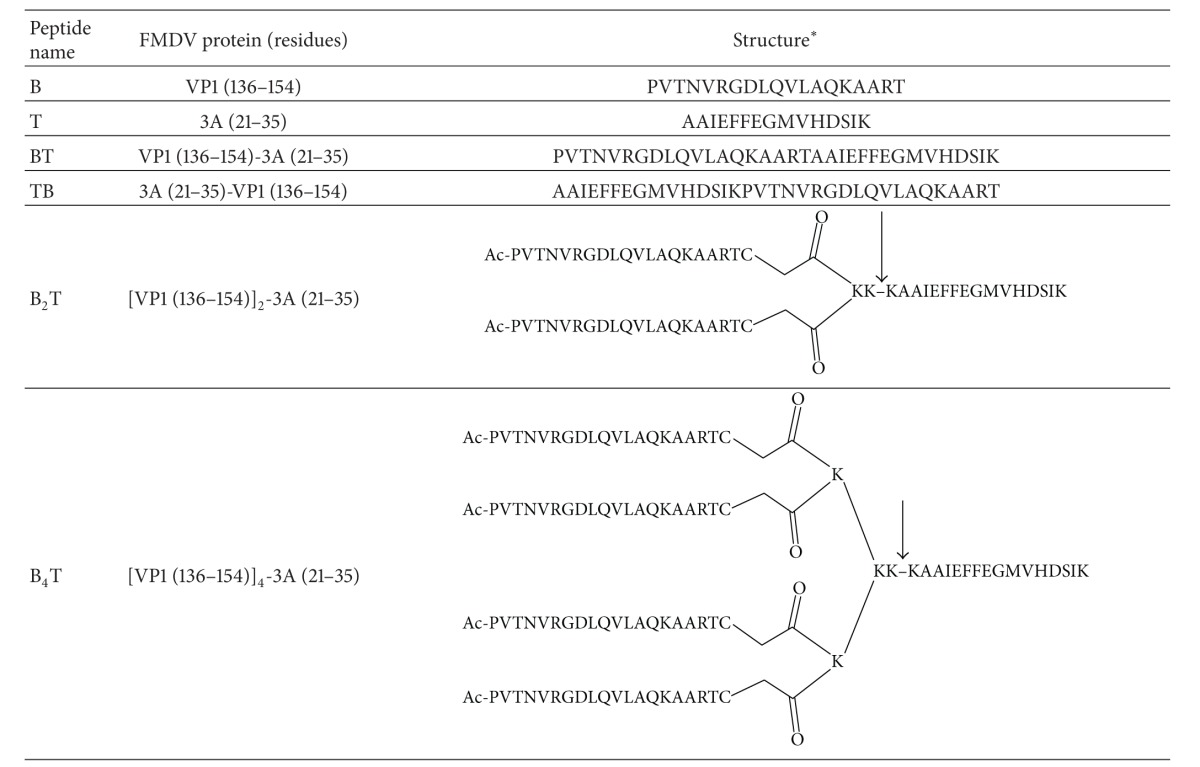

*All peptides in C-terminal carboxamide form. The arrow in B_2_T and B_4_T structures indicates a putative cathepsinD cleavage site [21].
